# Patient navigation for colorectal cancer screening in deprived areas: the COLONAV cluster randomized controlled trial

**DOI:** 10.1186/s12885-022-10169-3

**Published:** 2023-01-06

**Authors:** A. Bourmaud, Y. Benoist, F. Tinquaut, C. Allary, J. Ramone-Louis, M. Oriol, J. Kalecinski, V. Dutertre, N. Lechopier, M. Pommier, S. Rousseau, A. Dumas, P. Amiel, V. Regnier, V. Buthion, F. Chauvin

**Affiliations:** 1 Clinical Epidemiology Unit, Robert Debré Hospital, AP-HP, and INSERM CIC-EC 1426 and INSERM ECEVE 1123, University of Paris, 48 Bd Sérurier, 75 019 Paris, France; 2 Public Health Department, Hygée Centre, Lucien Neuwirth Cancer Institute, Saint Priest en Jarez, France and Inserm, Clinical Investigation Center 1408, 42055 Saint-Etienne, France; 3 Quality Safety Performance in Health (HESPER) EA7425, Lyon 1 University, Lyon, France; 4 Unité de Recherche en Sciences Humaines Et Sociales (URSHS) Institut Gustave Roussy, Paris, France; 5 COACTIS EA 4161 - Centre de Recherche en Gestion - Research Center in Management Science ISH and Faculty of Economics and Management, Lumière Lyon 2 University, Lyon, France; 6 Lyon1 University, Lyon, France; 7 UMR S2HEP, French Education Institute, Ecole Normale Supérieure de Lyon, Lyon, France

**Keywords:** Colorectal cancer screening, Patient navigation, Complex interventions, Health inequalities, Randomized control trial, Population health intervention research

## Abstract

**Background:**

The objective of this study was to assess the effectiveness of a Patient Navigation Intervention targeting deprived patients for Colo-Rectal Cancer (CRC) screening participation.

**Methods:**

A cluster randomized controlled trial was conducted in 5 districts. Peer Lay Patient Navigators were recruited to operate in deprived areas. Eligible participants had to be between 50 and 74 years old, live in these deprived areas and receive an invitation to the nationally organized Colo-Rectal Cancer (CRC) screening during the study period. The theory-driven navigation intervention was deployed for 18 months. A population Health Intervention Research assessment method was used to assess effectiveness and context interaction. The primary criterion was screening participation at 12 months.

**Results:**

Twenty-four thousand two hundred eighty-one individuals were included inside 40 clusters. The increase in participation in the intervention group was estimated at 23%, (ORa = 1.23, CI95% [1.07–1.41], *p* = 0.003). For the subgroup of individuals who participated, the time delay to participating was reduced by 26% (ORa = 0.74, CI95% [0.57–0.96], *p* = 0.021). Main factors modulating the effect of the intervention were: closeness of navigator profiles to the targeted population, navigators’ abilities to adapt their modus operandi, and facilitating attachment structure.

**Conclusion:**

The ColoNav Intervention succeeded in demonstrating its effectiveness, for CRC screening. Patient Navigation should be disseminate with broader health promotion goals in order to achieve equity in health care.

**Trial registration:**

clinicaltrials.gov NCT02369757 24/02/2015.

**Supplementary Information:**

The online version contains supplementary material available at 10.1186/s12885-022-10169-3.

## Background

Colo-rectal cancer (CRC) is the third leading cause of cancer deaths worldwide [1] and the second in France [2]. Screening has demonstrated its efficacy and efficiency in reducing CRC incidence and mortality rates over the past 2 decades among adults aged 50 years and older [3–5]. Until recently, international recommendations were to undergo regular screening with either a high‐sensitivity, stool‐based test or a structural (visual) examination, depending on patient preference and test availability, for all people (men and woman) aged between 50 and 75 [6]. In France, a population-based CRC screening program is in operation, targeting all French individuals aged from 50 to 74 without higher risk factors for CRC. This population is encouraged by mail to consult their general practitioner, who delivers the screening test. The test used until 2015 was the Fecal Occult Blood Test (FOBT) and since 2015 is the Fecal Immunochemical test (FIT). It is recommended to repeat this screening every two years. In order to maximize the health benefit and to be efficient, this screening program requires a participation rate of at least 50% [7, 8] which is constantly only above 40% [9]. Furthermore, it has been highlighted that the socio-economically deprived population participates less than others [10]. France is one of the European countries with the highest rate of social health inequalities, impacting mortality and cancer rates, including the CRC [11–13]. Factors associated with social inequalities in health are lower socio-economic status, income, educational level and health literacy level [14]; ethnic minorities participate less in CRC screening than their white counterparts, whichever factors are adjusted for [15, 16].

Patient Navigation (PN) interventions are programs specifically designed to overcome the individual’s perceived barriers to screening [17]. These programs are culturally tailored and target a specific deprived population in order to straighten the path to CRC screening for this population. Obstacles tackled by such programs range from unawareness of the existence of CRC screening to transportation issues, and include issues of belief or disbelief. These interventions have been increasingly adopted in the United States of America (USA) and Canada [18]. When this study began, Patient Navigation interventions had never been trialed in France, although an intervention targeting social inequities in health and aiming to improve CRC screening participation should be attractive in such a setting. This study was undertaken to assess the effectiveness of a PN program for CRC screening participation in France, among a deprived population, through a cluster randomized control trial. The secondary objectives were to assess the effect of the context on the effectiveness as well as on the mode of operation of Patient Navigators.

## Methods

### Study design

COLONAV is a cluster randomized controlled trial conducted in 5 French Departments (districts). It assesses the effectiveness of a Patient Navigation Intervention among deprived populations. The study used a complex interventions evaluation methodology to assess the effectiveness of this intervention [19].

The study was approved by an institutional review board (Ethical Committee of Saint Etienne University Hospital, 10^th^ of January 2013), which waived the need for signed informed consent according to French law, since the study involved healthy people and proposed no treatment. The study protocol has been detailed elsewhere [20].

In the framework of real world complex intervention evaluation [21–24] this study performed a multidisciplinary assessment, with mixed quantitative and qualitative methods, in order to assess, besides the efficacy for health outcomes, the effect of the context [25].

• Effectiveness for health outcomes was evaluated quantitatively through the cluster randomized control trial.
• Context evaluation was performed quantitatively through the strata analysis alongside the trial and qualitatively through an ethnographic evaluation.

### Cluster randomized control trial

#### Selection process

Five districts (départements) were selected according to their characteristics and their CRC screening participation rates (< 30%): five different districts with a low participation rate were retained: i) A rural district (Ardèche,) ii) A district with an industrial economy and low development (Loire) iii) A district with a tertiary economy and high development (Rhône) iv) A district where CRC screening has been implemented for a long time (Côte d’Or,) v) One of the suburban districts of Paris, highly urban (Val-de-Marne).

The cluster unit is the “Ilots Regroupés pour l'Information Statistique” (IRIS) zone, or « Clustered Units for Statistical Information» zone. Each district in France is divided into IRIS zones, ie aggregated units for statistical information used as a system for dividing the country into units of equal size (2000 residents per basic unit). IRIS zones eligible for this study were IRIS zones from the 5 districts selected for their low participation rate in CRC screening and with socioeconomic indicators of a deprived population. Deprived IRIS zones inside districts were selected using an ecological deprivation index. The French European Deprivation Index (EDI) was used to determine the socio-economic level of the population living in IRIS zones. French EDI is divided into 5 quintiles; from 1 (least deprived) to 5 (most deprived). For all 5 districts, IRIS zones with an EDI corresponding to a deprived population (score 4 and 5) were all selected for the randomization. The mean number of deprived IRIS zones by district was 384. A total of 1920 deprived IRIS zones were selected.

#### Participants

Participants included in the study were due to receive an invitation to the nationally organized Colo-Rectal Cancer (CRC) screening during the study period. Invitation is by an administrative letter sent every two years to those aged between 50 and 74 by a screening management structure. Invitations sent by the structure encourage people to consult their General Practitioner (GP) so that he delivers them the screening test, which is made at home and sent for analysis by mail to the laboratory. Results are sent back to the GP and the screening management structure, where the data were collected.

Study participants were men and women aged from 50 to 74, invited to participate in the CRC screening. The inclusion criteria were: to be between 50 and 74 years old at the time of selection, to be covered by health insurance, to have their postal address located within the IRIS zone concerned, and to be included in the register of persons eligible for organised colorectal cancer screening. Being included in this register led to an automatic invitation to screening every 2 years. The exclusion criteria were not being included in the registry because of a change of residence, lack of insurance coverage, or ineligibility for screening (history of colorectal cancer, or at high or very high risk of colorectal cancer, and therefore followed up elsewhere). Data on participation in screening in the 2 years prior to the start of the study were collected retrospectively from the screening management structures for all participants. Participants were followed prospectively for 2 years after the implementation of the intervention. 1 year during which the navigators carried out their intervention and 1 year for the collection of screening data.

#### Randomization and blinding procedures

Among all IRIS zones from the 5 districts eligible for the study (N = 1920), the participant IRIS were randomly selected with a 2 steps selection process. The centralized randomization was stratified by districts. For the experimental group, one IRIS per district was selected randomly from the eligible IRIS. Then 3 eligible adjacent IRIS were selected accordingly, in order to construct the experimental group. It was mandatory for the experimental group of 4 IRIS to have a continuous geographical setting, since the Navigator was supposed to act in a geographical “puddle-like” unit of action. For the Control group: 4 remote IRIS were randomly selected from the eligible IRIS of the district The randomization was performed at the IRIS level and was performed at once, before the beginning of the study, by an independent statistician. The allocation ratio of experimental and control groups was set at 1:1.

The trial was an open trial since no blinding was possible. But individuals not contacted by the navigator and individuals in the control group were blinded to their group, and even blinded to the study. Investigators were blinded to the measurement and collection of the primary outcome.

Sample size calculation estimated that the number of IRIS zones needed to meet the objectives of the study was 20 IRIS in the intervention group and 20 in the control group. This estimation relies on the following assumptions: We estimated that the intervention would be relevant if it provided an 8% absolute increase in the colorectal cancer screening participation rate. With a two- sided α risk of 0.05 and a β risk of 0.2, and assuming an intraclass correlation coefficient for clusters of 0.04 and a mean of 500 patients per cluster (assuming an average of 2000 people per IRIS zone and percentage of people between 50 and 75 years equal to 25% of the total population), we needed 20 clusters per arm. This represents: 4 experimental IRIS zones and 4 control IRIS zones per department. For a total of 40*500 = 20 000 patients).

### Intervention

#### Development of the intervention based on collected evidence

The first step of the Colonav study, was to investigate French local context characteristics and to tailor the patient navigation intervention to those characteristics. A field diagnosis was performed by a sociologist. Fifty-three interviews with professional stakeholders were conducted prior to the construction of the study. Details and results of this field diagnosis are given in supplementary material.

The Patient Navigation Intervention construction as well as Patient Navigators recruitment has been described in the protocol’s article [20]. The construction of the intervention relies on the Fiscella conceptual framework [26] where Patient Navigation in cancer care is defined as: “individualized assistance offered to patients, families, and caregivers to help overcome barriers and facilitate timely access to quality medical and psychosocial care from pre- diagnosis through all phases of the cancer experience”. The balance between the healthcare system and the community is at the root of this framework. Navigators recruitment and training had to follow field diagnosis recommendations. Finally, the PN had to have an institutional attachment, in order to ensure scientific legitimacy, as reported in the Fiscella model. The PN was to be hosted by the local team of the French National Patient Association Against Cancer (a Non-Governmental Organization, well established in France: La ligue Nationale contre le Cancer).

### Control

Men and women in the control group lived in the IRIS zones (clusters) randomized in the control group. They were aged 50 to 74 and the usual process for CRC screening was delivered to them, without changes.

### Effectiveness outcomes measures

#### Primary outcome

The primary outcome is the difference between before/after participation rates (12 months duration) in the intervention IRIS zones and in the control IRIS zones. This measure was collected directly from the cancer screening management structures of the 5 districts, so investigators, PN and subjects were end-point blinded.

#### Secondary outcomes

The time delay to participating was assessed according to the recommendations made by the National Patient Navigation Leadership Summit [26].

### Contextual analysis

#### Strata analysis

In order to capture the differential effectiveness of the PN interventions according to the context (PN characteristics, population and geographical characteristics) each primary and secondary outcome was assessed by district strata.

#### Ethnographic study

In order to capture the context influence on the PN mode of operation a qualitative analysis was performed by an ethnographer. Participants were the five navigators, and people targeted by the Colonav PN intervention, met by the navigator during the survey (3 days spent in immersion with the PN). This survey was based on qualitative methods: Observations were ethnographic in nature. These observations consisted on the one hand of semi-directive interviews and on the other hand of on-site observations. The latter were participatory in the sense that the researcher is not external to the action but interacts with the actors involved. Local involvement of the researcher was preferred to exteriority of vision. The data from on-site observations were recorded, in the most exhaustive way possible, in a field journal by the researcher. The interviews were semi-structured, conducted on the basis of an interview guide which indicates a list of themes. Themes were approached in the natural order of the conversation, taking into account the contingencies of the interaction. Part of the interview was devoted to the interviewee's life story and useful socio-demographic and socio-economic descriptions. The interviews were recorded (with the express oral agreement of the interviewee) and transcribed in full.

### Analysis

#### Statistical

All variables were described by frequency (%), with missing data, for categorical variables, and mean (SD) or median (Q1-Q3), with missing data, for quantitative variables. The effectiveness of the intervention was measured comparing the delta of before-after participation rate in the intervention arm and the control arm. The primary outcome (the difference between before/after participation rates) was analyzed by logistic regression analysis methods taking into account the interactions between the period and area. Completion time of the screening test between intervention areas and control areas before and after the intervention was also compared. In order to address cluster design issues, the influence of other collected variables was tested using univariate tests (Chi2 or Fisher test for categorical variables and Student or Wilcoxon test for quantitative variables). A multivariate model was implemented in order to take possible confounding factors into account. Analysis of delay to participation was performed with an ANOVA model (on the sub-group of participating patients). A priori strata analyses were performed for the same outcomes, with the same statistical methods, inside each stata.

All analyses were carried out using SAS version 9.3 and R software (version 3.2.2).

#### Ethnographic

The data were analyzed using the classical inductive methods of social anthropology. The strategy for analyzing observational and interview data combined the "case-oriented" and "variable-oriented" approaches: it involved both reporting on the functioning of the situation for itself and reporting on similarities and differences between situations on variables related to representations, health behaviors and situations experienced by the subjects. Each case, through the data collected on it, was analyzed in depth using thematic matrices; the transversal variables were gradually emergent. When data saturation was reached, final results were produced.

## Results

### Effectiveness analysis

**Fig. 1:**
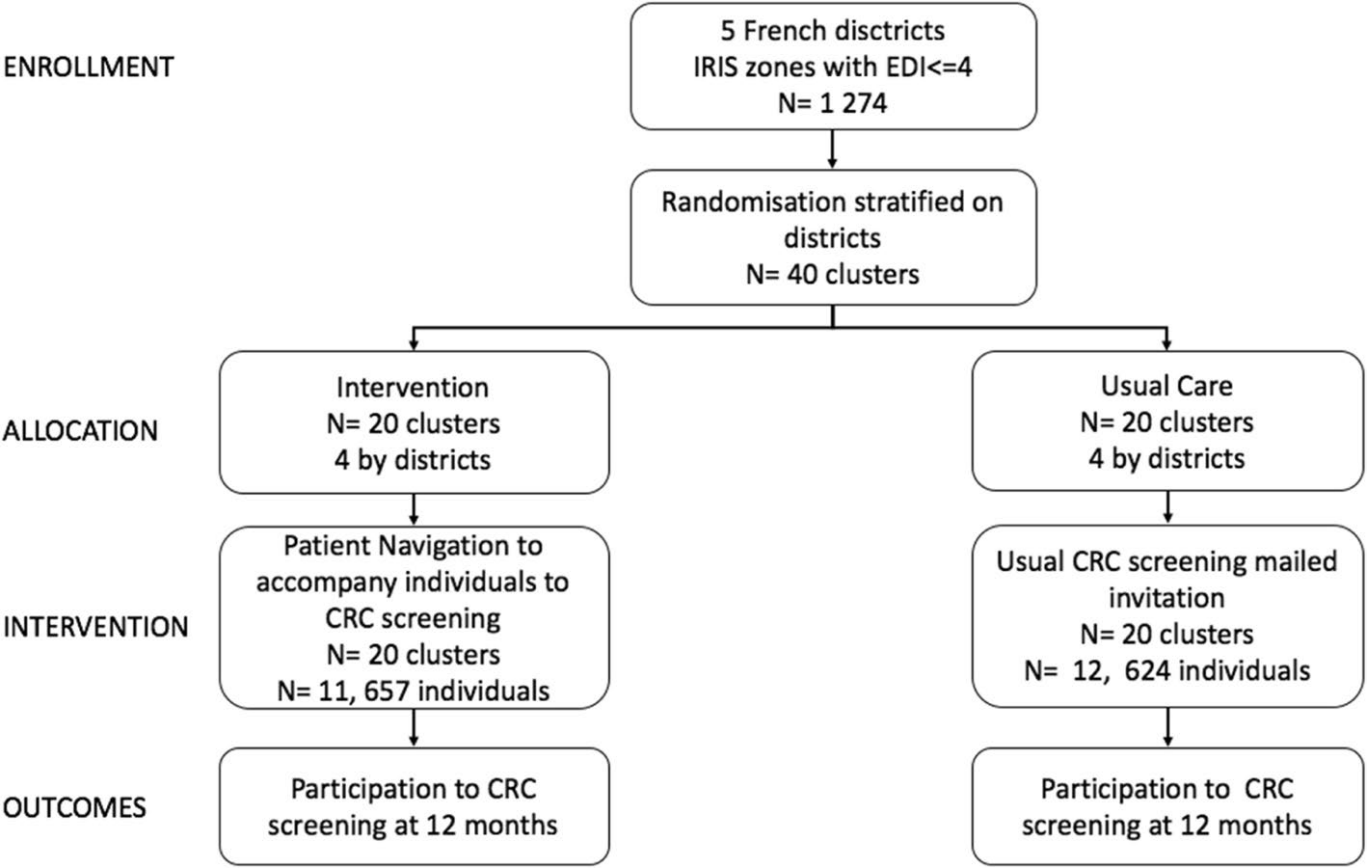
Study Flow Chart

Forty clusters were included in the study, from 5 different districts. 24,281 individuals living in the selected clusters were sent an invitation to participate in CRC screening during the study period (November 2014-November 2015), meaning that 24,281 subjects were included in this research, 12,624 in the control group and 11,657 in the intervention group (Fig. 1, Table 1). Their distribution by districts is representative of the specific population density of each of those districts. The majority of the subjects had never been screened for CRC (65,4%). The two groups were unbalanced in terms of age, gender and time since last participation for people having previously performed the screening.

**Table 1:** Patients’ characteristics for both arms

Variables	Modality	Control arm (*N* = 12,624)	PN Intervention arm (*N* = 11,657)	Total (*N* = 24,281)	*P* value
Gender	Female	6817 (54%)	6087 (52.2%)	12,904 (53.1%)	
	Male	5807 (46%)	5570 (47.8%)	11,377 (46.9%)	**0.006**
Age	Mean (sd)	61.3 (6.9)	61.2 (7.1)	61.2 (7)	0.155
	Min—max	49.3—76.1	50—75.1	49.3—76.1	
Age	(49,55]	2976 (23.6%)	2893 (24.8%)	5869 (24.2%)	
	(55,60]	2793 (22.1%)	2556 (21.9%)	5349 (22%)	**0.027**
	(60,65]	2714 (21.5%)	2352 (20.2%)	5066 (20.9%)	
	(65,70]	2400 (19%)	2181 (18.7%)	4581 (18.9%)	
	(70,75]	1722 (13.6%)	1660 (14.2%)	3382 (13.9%)	
	NA	19 (0.2%)	15 (0.1%)	34 (0.1%)	
District (Département)	Rhône-Alpes	3017 (23.9%)	3085 (26.5%)	6102 (25.1%)	
	Loire	2590 (20.5%)	3108 (26.7%)	5698 (23.5%)	** < 0.001**
	Côte d’or	2467 (19.5%)	2076 (17.8%)	4543 (18.7%)	
	Val de Marne	2604 (20.6%)	1761 (15.1%)	4365 (18%)	
	Ardèche	1946 (15.4%)	1627 (14%)	3573 (14.7%)	
Previoulsy screened	No	8259 (65.4%)	7616 (65.3%)	15,875 (65.4%)	0.373
	Yes	3003 (23.8%)	2847 (24.4%)	5850 (24.1%)	
	NA	1362 (10.8%)	1194 (10.2%)	2556 (10.5%)	
Time since last participation (for those previously screened) in months	Frequency	2254 (99.8%)	2325 (99.8%)	4579 (99.8%)	
	Mean (sd)	4.4 (3.3)	4.2 (3.4)	4.3 (3.4)	**0.007**
	Min—max	00—20.3	00—22.8	00—22.8	

**Fig. 2:**
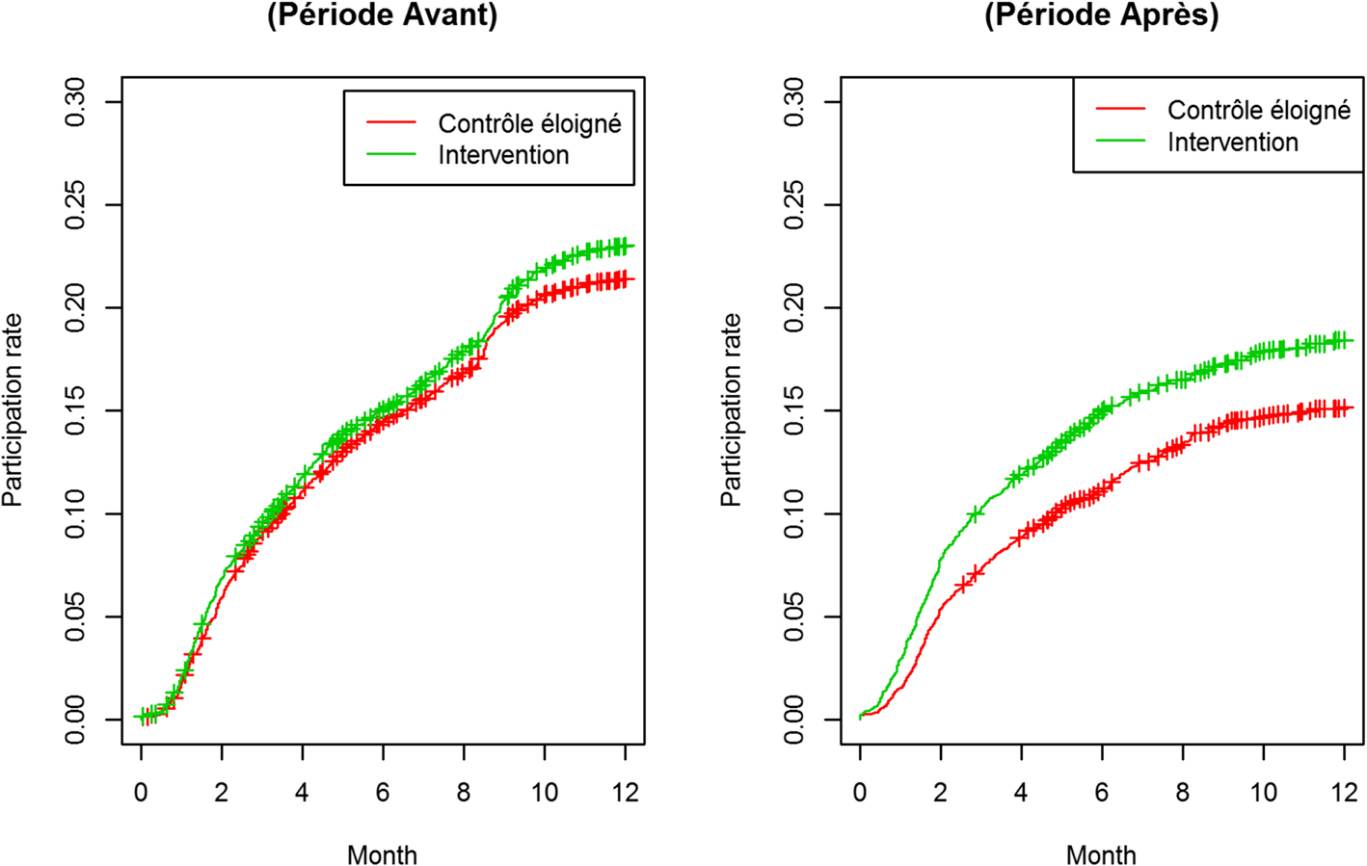
Evolution of participation rate by time (Kaplan Meier curves) according to the group, for the whole population of the trial

The results of this study (Fig. 2) show a participation rate of 21.4% in the control group in the 12 months prior to the implementation of the intervention and 21.9% in the experimental group in the same period. Following the implementation of the intervention in the intervention group territories, participation at 12 months was 14,6% in the control group and 18.6% in the experimental group. This corresponds to a crude increase of 2,5% in participation between the 2 groups and between the 2 periods, and a relative increase of 15,5%. The results on the primary criteria (Table 2, Fig. 2) highlighted a statistically significant effect of the patient navigation intervention on the CRC screening participation rate at 12 months, with an increase of participation between both groups of 15% (OR = 1.15, CI95% [1.01,1.31], *p* = 0.038). The difference in difference (DID) model that was chosen to estimate the effect of the PN intervention reports: data on allocation of groups, and time periods. In such a model, it is the coefficient on the interaction term that is an estimate of the intervention effect under the common trend assumption. The size of the effect of the intervention on the participation rate is increased when adjusting for unbalanced variables (age and gender) and districts (Table 3), with the DID model: it is estimated at 23% (ORa = 1.23, CI95% [1.07,1.41], *p* = 0.003). The time delay to participation for the subgroup of people who participated in screening was, prior to the implementation of the study, a median of 3.8 months (IQR 2.0–6.5) for the control group and 3.7 months (IQR 1.8–6.8) for the intervention group. This drops to a median of 3 months (IQR 1.7–5.3) for the control group and 2.5 months (IQR 1.6–4.7) for the experimental group, i.e. an accelerated participation of 0,5 month for the experimental group compared to the control group. The time delay to participation (table S1 supplementary material) was statistically significantly reduced inside the intervention group, after adjustment for age, gender and districts: it decreased by 26% (OR = 0.74, CI95% [0.57,0.96], *p* = 0.021).

**Table 2:** primary outcome assessment with logistic regression and interaction

*N* = 24,281	Crude OR (95%CI)	Adj. OR (95%CI)	*P* (Wald's test)
Time: after vs before	0.72 (0.67,0.77)	0.67 (0.61,0.73)	< 0.001
Arm: Intervention vs Control	1.15 (1.07,1.22)	1.09 (1.01,1.19)	0.035
Time*arm = Intervention effect	-	**1.15 (1.01,1.31)**	**0.038**

Legend: the effect of the intervention is estimated by the interaction between time and arm, with an adjusted Odds Ratio.

**Table 3:** multivariate analysis of the intervention effect, ajusted on confusing variables ( age districts and gender)

*N* = 24,281	Crude OR (95%CI)	Adj. OR (95%CI)	*P* (Wald's test)
Time: after vs before	0.72 (0.67,0.77)	0.7 (0.64,0.78)	< 0.001
Arm: Intervention vs Control	1.15 (1.07,1.22)	0.999 (0.919,1.087)	0.975
Districts ref = 21			
42	0.91 (0.83,1)	0.89 (0.81,0.97)	0.008
69	0.62 (0.57,0.68)	0.61 (0.55,0.67)	< 0.001
7	0.52 (0.46,0.58)	0.53 (0.48,0.6)	< 0.001
94	0.09 (0.07,0.1)	0.09 (0.07,0.11)	< 0.001
Age:]53–70] vs [49–52]	1.28 (1.16,1.4)	1.33 (1.2,1.46)	< 0.001
Gender: M vs F	0.74 (0.69,0.79)	0.77 (0.72,0.82)	< 0.001
Time*arm = **intervention effect**	-	**1.23 (1.07,1.41)**	**0.003**

Legend: the effect of the intervention is estimated by the interaction between time and arm, with an adjusted Odds

### Context analysis

The strata analysis did not identify any significant efficacy of the patient navigation intervention at the district level, even after adjustment for age and gender (tables S2 to S6, in supplementary material).

**Fig. 3:**
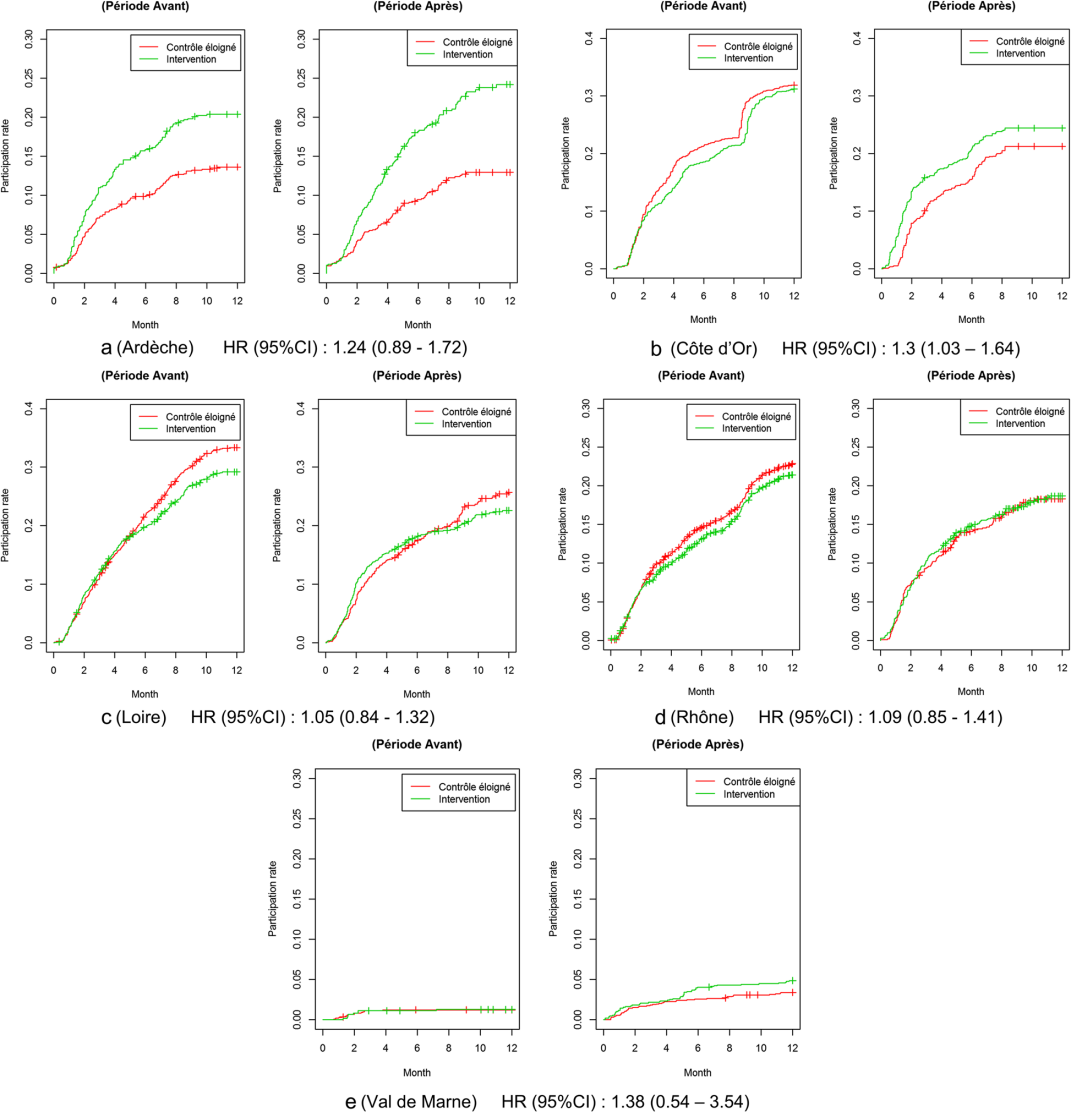
Strata analyses: evolution of participation rate by time (Kaplan Meier curves) according to the group by districts. **a** (Ardèche) HR (95%CI): 1.24 (0.89—1.72). **b** (Côte d’Or) HR (95%CI): 1.3 (1.03 – 1.64). **c** (Loire) HR (95%CI): 1.05 (0.84—1.32), **d** (Rhône) HR (95%CI): 1.09 (0.85—1.41). **e** (Val de Marne) HR (95%CI): 1.38 (0.54 – 3.54)

However, figures for participation evolution over time according to time and groups display a heterogenous process of participation according to district and also according to patient navigator (Fig. 3). In Ardeche, cumulative participation shows a marked increase in the participation rate between before and after inside the intervention group but a marked decrease of participation inside the control group. The before-participation rate at 12 months was of 13.4% in the control group and 20.3% in the experimental group. The after-participation rate at 12 months was 12.8% in the control group and 23.9% in the experimental group i.e. a crude increase of 4.5%. The increase in participation was estimated at 23% (adjusted OR, *p* = 0.27) by the DID model, but without statistical significance. In the Côte d’Or, both participation rates decreased between before and after, but there was a markedly smaller decrease in the intervention group than in the control group. The before-participation rate at 12 months was of 32.1% in the control group and 31.2% in the experimental group. The after-participation rate at 12 months was 21.4% in the control group and 24.5% in the experimental group i.e. a crude increase of 3.9%. This led to an increase in favor of the intervention group, but still not significant (+ 17%, adjusted OR, *p* = 0.24). In the Loire district and in the Rhône district, both participation rates decreased between before and after, but remained very similar between both groups, although the global decrease was bigger in the Loire than in the Rhône district. In the Loire district the before-participation rate at 12 months was of 33% in the control group and 28.7% in the experimental group. The after-participation rate at 12 months was 25.3% in the control group and 22.3% in the experimental group i.e. a crude increase of 1.3%. In the Rhône district the before-participation rate at 12 months was of 22.8% in the control group and 21.4% in the experimental group. The after-participation rate at 12 months was 18.3% in the control group and 18.6% in the experimental group i.e. a crude increase of 1.7%. In both districts the results of the DID models showed a slight increase in favor of the intervention group: 12% more (adjusted OR, *p* = 0.37) for the Loire district and 8% more (adjusted OR, *p* = 0.61) for the Rhône district. In the Val de Marne, the baseline participation rate was very low in both groups, compared to other districts. Here, the before-participation rate at 12 months was of 1.25% in both control and intervention group. The after-participation rate at 12 months was 3.1% in the control group and 4.7% in the experimental group i.e. a crude increase of 1.6%. In this district, participation rates increased between the before and the after period in both groups, with a larger increase in the intervention group, estimated by the DID model, but not significantly (+ 12%, adjusted OR, *p* = 0.8).

Context interactions were explored through the ethnographic analysis. Navigators identified the main characteristic of their intervention area as the predominance of poverty among the people encountered. This poverty puts CRC screening far behind in the order of these people's priorities. Since the targeted population was the deprived population, the poverty barriers were all the more present. The navigators first had to discuss and propose solutions about bills, rents, children's job search and problems with the justice system. Another main contextual characteristic underlined was the language: navigators encountered many people who did not speak French. Navigators sharing a common language with the local communities were more adapted to their function, since they manage to lower this barrier. Another contextual factor that affected navigators’ actions was the behavior of the institutions supposed to give navigators legitimacy: some were helpful and opened doors for him/her, while some were real obstacles for the navigation. Navigators had to develop two contextual modes of action to establish first contact with people and to address those issues: Navigators from Loire, Rhône and Côte d’Or adopted a strategy of direct contact. This because the 3 navigators were already integrated in the intervention zone before the study. They were already known by a part of the population they were meeting. On the other hand, the difficulty was to be recognized as professionals: all 3 of them had to approach an institution frequented by the inhabitants, which made it possible to legitimize their function. Subsequently, they came into contact with the population in various places of daily life (markets, mosque, bus stop). From the very first meeting they started discussing screening. The other strategy was that of the Val de Marne and Ardeche navigators. they were both foreign to their field of action before the study began. They needed to gain the trust of the population, and to do so, they needed to become visible. They felt that a direct contact could be considered intrusive and therefore counterproductive. One of the navigators ensured visibility by first helping NGO volunteers (meal distribution). He spoke of CRC screening only when he was well known by the people. The other one was the only navigator who had real difficulties in reaching the targeted population. His strategy was to hold permanent meetings and organize workshops. He presented the test to the people who came to meet him, never the other way around. No official institution helped him.

From these broad contextual characteristics each navigator invented her/his own original operating procedures. The populations in the intervention areas were all heterogeneous and therefore a community approach was difficult to apply. As a result, navigators favored the recruitment of part of the population, depending on their ethnicity or social level. Patients Navigators developed their mode of operation on the model of the “bricoleur “ (Do It Yourself building) in the sense of C. Levi-Strauss [27] (" the universe of the Bricoleur is closed, and he often is forced to make do with whatever is at hand”, so that the bricoleur is able to perform a large number of diversified tasks). Navigators-Bricoleurs identify the tools in their environment and imagine how they will be useful to them. The skill of the navigator-bricoleur is to use the most appropriate tool in the most appropriate way for the situation.

## Discussion

This study found a large and statistically significant effect of the Colonav Patient Navigation Program on deprived populations’ participation rate in CRC screening. This effect was estimated as an increase of 23% in participation in the population receiving the intervention. In addition, this study explored more broadly the interaction between the context and the intervention deployment.

The quantitative context analysis highlights the differences among the districts at the beginning of the study. Initial participation rates ranged from 30 to 0%. Even with such different contexts at the beginning of the intervention, the study underlines a positive (but not significant) effect of each of the navigators. The context analysis underlined that main contextual factors identified as impacting the navigators’ mode of operation were: poverty (a major barrier to keeping healthy), language barrier (categorised as present or not) and behavior of the institutions supposed to give the navigators legitimacy (supportive or opposed). In this, our study confirms Ficella's postulate on the PN, which implies a double recognition: that of peers but also that of Institution. On the other hand, the COLONAV study broadens the description of the mechanism implemented by navigation: It highlights that the low socio-economic level of certain populations, particularly in urban districts, was a ubiquitous feature, and shaped a part of the navigators’ mode of operation: here they had to spend much time in first resolving the social problems of individuals, before starting to accompany residents to CRC screening. But this problem-solving aspect was a mandatory step towards success in their action. Depending on their previous knowledge of the population (same culture, same place of residence, same language) navigators had different levels of facilities for approaching them, and also had different intrinsic abilities. So they developed different strategies to reach people, each with its own strengths and weaknesses. The common feature was that they all relied on the existing social network and that they had to adapt and be flexible to fit with their unpredictable and moving environment. The essence of Navigation is here to make do with what is already there. It does not establish a position of its own but is a link between the already existing systems.

Our results are consistent with the broad range of other published PN studies. effect of PN for CRC screening in USA has been estimated at OR = 2.01 IC95% (1.64–2.26) [28]. The only other French study assessed navigators who telephoned to deprived non-participants to enhance their participation in CRC screening [29]. The effect of the PN was estimated at an increase of 19% in participation. But the PN intervention was standardized (phone call procedures) unlike the Colonav study where PN were empowered to construct their job, to adapt it to the context and to their intrinsic characteristics. This study thus demonstrates the tremendous feasibility and operationality of broad PN.

The Cluster randomized design is another strength of this study, since it is adapted to population-based research, allowing it to avoid contamination bias. Another strength is the size of this study: 24,281 individuals were included, across 5 different French districts. The study included 4 different research teams from 5 disciplines. With such a large scale, the study succeeded in finding significant proof of the effectiveness of a French Patient Navigation Intervention for CRC screening among a deprived population.

The study also had limitations. The first was the unfortunate timing of the course of the study, which was conducted at the same time as France changed the type of test for CRC screening from FOBT to Fecal Immunochemical Test (FIT). We had to face, in the middle of the study, a period where no tests of any kind were available. This explains the drop in participation rate between the before and the after comparison. Fortunately, the existence of control groups mitigated this effect in the results. And the effect of PN in those worst conditions remained positive, which increases the possibility of a greater effect under better conditions. The second limitation was the impossibility of having access to individual data for the target population. However, we believe that Population Health Intervention Research is by its essence ecological research. Qualitative analyses provide necessary and complementary information which is lacking in quantitative findings.

## Conclusion

The Colonav study succeeded in demonstrating the effectiveness of Patient Navigation in France, for CRC screening. This is major step since PN interventions target deprived populations and France is one of the European countries with the highest socio-economic gradient. New studies should investigate this PN model, for other cancer screenings and broader health promotion goals, in order for health care equity to be achieved.

## Supplementary Information

**Additional file 1:** **Table S1.** Multivariate analysis of the effect of the intervention on time to participate in the participating sub-group, with an ANOVA model adjusted on confusing variables (age, district and gender ). **Table S2.** Ardèche. **Table S3.** Côte d’Or. **Table S4.** Loire. **Table S5.** Rhône. **Table S6.** Val de Marne.

## Data Availability

The datasets generated and analysed during the current study are available from the corresponding author on reasonable request.
