# Supplementary material for: Patient navigation for colorectal cancer screening in deprived areas: the COLONAV cluster randomized controlled trial
Source: BMC Cancer. 2023 Jan 6;23:21. doi: 10.1186/s12885-022-10169-3 (PMC9817361; doi:10.1186/s12885-022-10169-3)
Supplement: Supplementary file 1 — Additional file 1: Table S1. Multivariate analysis of the effect of the intervention on time to participate in the participating sub-group, with an ANOVA model adjusted on confusing variables (age, district and gender ). Table S2. Ardèche. Table S3. Côte d’Or. Table S4. Loire. Table S5. Rhône. Table S6. Val de Marne. [file 12885_2022_10169_MOESM1_ESM.docx]

Supplementary Material

PN INTERVENTION

*Development of the intervention based on collected evidence:*

A field diagnosis was performed by a sociologist, in order to identify the issues, levers and obstacles to the implementation of the Colonav project; to contribute to the definition of the navigator function; thus to build the Colonav intervention tailored to the field, the actors, the practices and the targeted population. An exploratory qualitative study was performed to do so. Fifty three interviews were conducted prior to the construction of the study ( January to July 2013). General practitioners, local elected officials, population representatives, prevention workers, representatives of non-governmental organisations were interviewed, in each district. French and local particularities identified in this qualitative study were: i) the difficulties of access to care for local people, due to the departure of healthcare professionals from disadvantaged areas ii) people with a particular relationship to the national health service, often isolated from it. The study found a population of diverse geographical, cultural and religious origins, resulting in singularities of belief (fatalism), language (communication problems), and isolation (ageing, rural location) iii) difficulties in accepting CRC screening as it is organised in France (the receipt of a paper invitation by mail, the need to test oneself at home, the taboo of stool collection, the absence of a positive relay by GPs). These contextual specificities were taken into account to construct the core requirements of the COLONAV Patient Navigation Intervention : i) the Patient Navigator (PN) should build her or her own intervention, choose relevant and original meeting places, encourage cooperation between actors, innovate in terms of the modality of approach, taking into account the facilities and different obstacles relating to lifestyles, physical and social environment and people's needs and dispositions; the PN should adapt his/her communication media to the audience ii) the PN should create a secure framework and assure the public of his/her legitimacy and competence by announcing their institutional affiliation iii) the PN should communicate with a comprehensive approach, in contrast to a normed and standardized approach, which focuses as a first step on CRC screening : the PN should, after community-based support work, identify the specific individual barriers that prevent the person from participating in the screening, help him/her to remove these barriers and accompany him/her to the screening iv) the PN should reflect a belonging to the group : approximatively same age, same origins, same culture, same languages, same experiences, living in the area. The PN should demonstrate a high plasticity in the fields of activities.

These elements were taken into account in the PN recruitment process and in the PN training content.

*COLONAV Patient navigation intervention : PN recruitment and training*

According to the results of the field diagnosis, recruitment forms were published and, in each district, a PN was recruited. Applicants were to share a common language, were to live (or to have lived) in the area of the cluster, to have experience in social work and communication and adaptation skills, and to be, as far as practically possible, older persons. possible. The five PN were then trained to perform their future function

PNs were given knowledge about CRC screening in France, about health disparities, about the research project ( objectives, stakes, expected benefit) , about the diversity of representations of the body and disease according to social and cultural groups. Then the expected function of PN was introduced to them : the main missions, responsibilities, activities, the expectations and objectives (facilitate access to screening: information, awareness-raising, support for the population). They were informed of what was outside the PN area of expertise (limits of the position). The ethical and educational support posture of the PN was discussed. The future PN were trained in how to identify relevant stakeholders according to context, how to communicate accordingly, how to encourage collaborations, and how to create a mapping framework.

RESULTS

**Table S1 : Multivariate analysis of the effect of the intervention on time to participate in the participating sub-group, with an ANOVA model adjusted on confusing variables (age, district and gender )**

| **N = 4455** | **crude OR(95%CI)** | **adj. OR(95%CI)** | **P(Wald's test)** |
| --- | --- | --- | --- |
|  |  |  |  |
| arm: Intervention vs Control | 0.8969 (0.7958,1.0108) | 1.0011 (0.8617,1.163) | 0.989 |
|  |  |  |  |
| time: after vs before | 0.63 (0.55,0.71) | 0.74 (0.61,0.88) | < 0.001 |
| Age  ref.=(49,55] |  |  |  |
| (55,60] | 0.95 (0.79,1.14) | 0.96 (0.8,1.15) | 0.628 |
| (60,65] | 0.89 (0.74,1.07) | 0.89 (0.74,1.07) | 0.233 |
| (65,70] | 0.64 (0.53,0.78) | 0.65 (0.54,0.78) | < 0.001 |
| (70,75] | 0.56 (0.45,0.69) | 0.55 (0.45,0.68) | < 0.001 |
|  |  |  |  |
| gender: M vs F | 1.17 (1.04,1.32) | 1.19 (1.05,1.35) | 0.005 |
|  |  |  |  |
| Time*arm  = **intervention effect** | - | **0.74 (0.57,0.96)** | **0.021** |
|  |  |  |  |

Strata analysis

**Table S2 : Ardèche**

| **N = 3568** | **crude OR(95%CI)** | **adj. OR(95%CI)** | **P(Wald's test)** | **P(LR-test)** |
| --- | --- | --- | --- | --- |
| time: after vs before | 0.95 (0.79,1.13) | 0.84 (0.64,1.11) | 0.217 | 1 |
|  |  |  |  |  |
| arm: Intervention vs Control | 1.85 (1.54,2.21) | 1.65 (1.27,2.15) | < 0.001 | 1 |
|  |  |  |  |  |
| Age  ref.=(49,55] |  |  |  | < 0.001 |
| (55,60] | 1.78 (1.4,2.27) | 1.79 (1.4,2.28) | < 0.001 |  |
| (60,65] | 1.07 (0.82,1.41) | 1.11 (0.84,1.46) | 0.464 |  |
| (65,70] | 0.66 (0.51,0.87) | 0.67 (0.51,0.89) | 0.005 |  |
| (70,75] | 0.54 (0.38,0.77) | 0.57 (0.4,0.8) | 0.001 |  |
|  |  |  |  |  |
| Gender: M vs F | 0.79 (0.66,0.95) | 0.76 (0.63,0.91) | 0.004 | 0.003 |
|  |  |  |  |  |
| Time*arm  = **intervention effect** | - | 1.23 (0.85,1.76) | 0.274 | 0.274 |
|  |  |  |  |  |

**Table S3 : Côte d’Or**

| **N = 4540** | **crude OR(95%CI)** | **adj. OR(95%CI)** | **P(Wald's test)** | **P(LR-test)** |
| --- | --- | --- | --- | --- |
| time: after vs before | 0.58 (0.51,0.66) | 0.53 (0.44,0.63) | < 0.001 | 1 |
|  |  |  |  |  |
| arm: Intervention vs Control | 1.06 (0.93,1.21) | 1.02 (0.85,1.21) | 0.854 | 1 |
|  |  |  |  |  |
| Age: ref.=(49,55] |  |  |  | < 0.001 |
| (55,60] | 1.02 (0.84,1.26) | 0.99 (0.8,1.21) | 0.906 |  |
| (60,65] | 1.4 (1.15,1.71) | 1.38 (1.13,1.69) | 0.002 |  |
| (65,70] | 1.46 (1.19,1.79) | 1.48 (1.21,1.83) | < 0.001 |  |
| (70,75] | 1.64 (1.31,2.06) | 1.77 (1.41,2.23) | < 0.001 |  |
|  |  |  |  |  |
| gender: M vs F | 0.74 (0.65,0.85) | 0.71 (0.62,0.81) | < 0.001 | < 0.001 |
|  |  |  |  |  |
| Time*arm  = **intervention effect** | - | 1.17 (0.9,1.53) | 0.249 | 0.249 |
|  |  |  |  |  |

**Table S4 Loire**

| **N = 5685** | **crude OR(95%CI)** | **adj. OR(95%CI)** | **P(Wald's test)** | **P(LR-test)** |
| --- | --- | --- | --- | --- |
| time: after vs before | 0.75 (0.66,0.86) | 0.71 (0.59,0.86) | < 0.001 | < 0.001 |
|  |  |  |  |  |
| arm: Intervention vs Control | 0.87 (0.77,0.97) | 0.85 (0.73,0.98) | 0.028 | 1 |
|  |  |  |  |  |
| Age: ref.=(49,55] |  |  |  | 0.004 |
| (55,60] | 1.25 (1.04,1.5) | 1.22 (1.01,1.47) | 0.038 |  |
| (60,65] | 1.36 (1.13,1.63) | 1.33 (1.11,1.6) | 0.002 |  |
| (65,70] | 1.41 (1.17,1.71) | 1.39 (1.15,1.68) | < 0.001 |  |
| (70,75] | 1.35 (1.11,1.65) | 1.33 (1.09,1.62) | 0.005 |  |
|  |  |  |  |  |
| Gender: M vs F | 0.85 (0.75,0.96) | 0.86 (0.76,0.97) | 0.017 | 0.017 |
|  |  |  |  |  |
| Time*arm  = **intervention effect** | - | 1.12 (0.87,1.45) | 0.371 | 0.371 |
|  |  |  |  |  |

**Table S5 Rhône**

| **N = 6096** | **crude OR(95%CI)** | **adj. OR(95%CI)** | **P(Wald's test)** | **P(LR-test)** |
| --- | --- | --- | --- | --- |
| time: after vs before | 0.86 (0.75,0.99) | 0.8 (0.66,0.98) | 0.03 | 1 |
|  |  |  |  |  |
| arm: Intervention vs Control | 0.97 (0.86,1.11) | 0.96 (0.82,1.12) | 0.606 | 1 |
|  |  |  |  |  |
| Age: ref.=(49,55] |  |  |  | < 0.001 |
| (55,60] | 1.7 (1.4,2.07) | 1.7 (1.4,2.06) | < 0.001 |  |
| (60,65] | 1.66 (1.36,2.02) | 1.64 (1.35,2) | < 0.001 |  |
| (65,70] | 1.98 (1.62,2.41) | 1.97 (1.62,2.41) | < 0.001 |  |
| (70,75] | 1.41 (1.13,1.75) | 1.42 (1.15,1.77) | 0.001 |  |
|  |  |  |  |  |
| Gender: M vs F | 0.71 (0.63,0.81) | 0.72 (0.63,0.82) | < 0.001 | < 0.001 |
|  |  |  |  |  |
| Time*arm  = **intervention effect** | - | 1.08 (0.81,1.42) | 0.612 | 0.612 |
|  |  |  |  |  |

**Table S6 Val de Marne**

| **N = 4358** | **crude OR(95%CI)** | **adj. OR(95%CI)** | **P(Wald's test)** | **P(LR-test)** |
| --- | --- | --- | --- | --- |
| time: after vs before | 3.85 (2.47,6) | 3.74 (2.1,6.67) | < 0.001 | 1 |
|  |  |  |  |  |
| arm: Intervention vs Control | 1.48 (1.05,2.07) | 1.17 (0.51,2.69) | 0.714 | 1 |
|  |  |  |  |  |
| Age: ref.=(49,55] |  |  |  | 0.292 |
| (55,60] | 0.74 (0.46,1.19) | 0.63 (0.39,1.02) | 0.059 |  |
| (60,65] | 0.69 (0.42,1.14) | 0.62 (0.38,1.03) | 0.068 |  |
| (65,70] | 0.84 (0.5,1.41) | 0.75 (0.44,1.26) | 0.273 |  |
| (70,75] | 0.73 (0.39,1.37) | 0.6 (0.32,1.13) | 0.112 |  |
|  |  |  |  |  |
| Gender: M vs F | 0.55 (0.39,0.78) | 0.55 (0.39,0.78) | < 0.001 | < 0.001 |
|  |  |  |  |  |
| Time*arm  = **intervention effect** | - | 1.12 (0.45,2.79) | 0.81 | 0.809 |
|  |  |  |  |  |
